# *sox9b* Is a Key Regulator of Pancreaticobiliary Ductal System Development

**DOI:** 10.1371/journal.pgen.1002754

**Published:** 2012-06-14

**Authors:** Marion Delous, Chunyue Yin, Donghun Shin, Nikolay Ninov, Juliana Debrito Carten, Luyuan Pan, Taylur P. Ma, Steven A. Farber, Cecilia B. Moens, Didier Y. R. Stainier

**Affiliations:** 1 Department of Biochemistry and Biophysics, Program in Developmental and Stem Cell Biology, Liver Center and Diabetes Center, University of California San Francisco, San Francisco, California, United States of America; 2 Department of Biology, Johns Hopkins University, Baltimore, Maryland, United States of America; 3 Department of Embryology, The Carnegie Institution for Science, Baltimore, Maryland, United States of America; 4 Howard Hughes Medical Institute and Division of Basic Science, Fred Hutchinson Cancer Research Center, Seattle, Washington, United States of America; University of Pennsylvania School of Medicine, United States of America

## Abstract

The pancreaticobiliary ductal system connects the liver and pancreas to the intestine. It is composed of the hepatopancreatic ductal (HPD) system as well as the intrahepatic biliary ducts and the intrapancreatic ducts. Despite its physiological importance, the development of the pancreaticobiliary ductal system remains poorly understood. The SRY-related transcription factor SOX9 is expressed in the mammalian pancreaticobiliary ductal system, but the perinatal lethality of *Sox9* heterozygous mice makes loss-of-function analyses challenging. We turned to the zebrafish to assess the role of SOX9 in pancreaticobiliary ductal system development. We first show that zebrafish *sox9b* recapitulates the expression pattern of mouse *Sox9* in the pancreaticobiliary ductal system and use a nonsense allele of *sox9b*, *sox9b^fh313^*, to dissect its function in the morphogenesis of this structure. Strikingly, *sox9b^fh313^* homozygous mutants survive to adulthood and exhibit cholestasis associated with hepatic and pancreatic duct proliferation, cyst formation, and fibrosis. Analysis of *sox9b^fh313^* mutant embryos and larvae reveals that the HPD cells appear to mis-differentiate towards hepatic and/or pancreatic fates, resulting in a dysmorphic structure. The intrahepatic biliary cells are specified but fail to assemble into a functional network. Similarly, intrapancreatic duct formation is severely impaired in *sox9b^fh313^* mutants, while the embryonic endocrine and acinar compartments appear unaffected. The defects in the intrahepatic and intrapancreatic ducts of *sox9b^fh313^* mutants worsen during larval and juvenile stages, prompting the adult phenotype. We further show that Sox9b interacts with Notch signaling to regulate intrahepatic biliary network formation: *sox9b* expression is positively regulated by Notch signaling, while Sox9b function is required to maintain Notch signaling in the intrahepatic biliary cells. Together, these data reveal key roles for SOX9 in the morphogenesis of the pancreaticobiliary ductal system, and they cast human *Sox9* as a candidate gene for pancreaticobiliary duct malformation-related pathologies.

## Author Summary

The liver and pancreas function as exocrine glands that secrete bile and pancreatic juice, respectively, to aid the digestion and absorption of nutrients. These fluids reach the intestine via the pancreaticobiliary ductal system, a complex network of ducts. Despite its pivotal role, the development of this ductal system is poorly understood. We have discovered that the zebrafish transcription factor gene *sox9b*, like its mammalian ortholog, is specifically expressed in the pancreaticobiliary ductal system. The perinatal lethality of *Sox9* heterozygous mice makes the analysis of SOX9 function challenging; thus, we turned to the zebrafish to analyze the role of SOX9 in pancreaticobiliary ductal system development. We found that zebrafish *sox9b* mutants, which survive to adulthood, display defects in the morphogenesis of this ductal network: the intrahepatic and intrapancreatic ducts fail to form a branched network, whereas the ducts connecting the liver and pancreas to the intestine are malformed. These ductal defects affect bile transport and lead to cholestasis in adult mutant fish. At the molecular level, Sox9b interacts with the Notch signaling pathway to regulate the development of the intrahepatic biliary network. Therefore, our work in zebrafish reveals a broad and complex role for SOX9 in pancreaticobiliary ductal system morphogenesis.

## Introduction

The pancreaticobiliary ductal system refers to the complex network of ducts that compose the hepatopancreatic ductal (HPD) system as well as the intrapancreatic and intrahepatic ductal networks. The HPD system consists of the extrahepatic duct, cystic duct, gallbladder, common bile duct, and extrapancreatic duct. It connects to the intrahepatic biliary ducts to enable bile flow and storage. The intrapancreatic ducts collect the digestive enzymes secreted by the pancreatic acinar cells. Pancreatic juice and bile flow to the hepatopancreatic ampulla to be released into the intestine and allow digestion and absorption of nutrients [1]. Malformations of the pancreaticobiliary ductal system impair the function of digestive organs and are associated with various congenital conditions whose causes are mostly unknown.

In mammals, the transcription factor Sox17 is specifically expressed in a segment of the ventral foregut from which the pancreaticobiliary ductal system derives [2]. This factor has been shown to be a master regulator of pancreaticobiliary ductal system formation by specifying, in conjunction with Hhex and Pdx1, different lineages of the liver, pancreas and HPD system [2]. The liver is specified as a group of cells that expresses Hhex but not Sox17 or Pdx1. The intrahepatic biliary network requires several signaling pathways including TGFβ, Notch and Wnt, to differentiate and mature (for a review, see [3]). In particular, Notch signaling has been shown to promote intrahepatic biliary differentiation and tubulogenesis [4]–[9]. Adjacent to the liver domain, cells expressing both Sox17 and Pdx1 delineate a domain that gives rise to the HPD system and pancreas [2]. After lineage segregation, Sox17+/Pdx1− cells give rise to the HPD system under the regulation of downstream factors such as HNF6, HNF1β and Hhex [2] which themselves have been shown to play important roles in the development of the HPD system [10]–[12]. As for the intrapancreatic ducts, they arise from a subset of Pdx1+/Sox17− cells that also express HNF6 and HNF1β [13], [14]. These two transcription factors regulate duct tubulogenesis as well as the differentiation of the epithelial cells lining the ducts [14].

The HPD system in zebrafish is morphologically similar to the one in amniotes. As in mammals, the zebrafish HPD system develops from a specific domain within the foregut endoderm that lies between the emerging liver and ventral pancreas [15]. HPD system patterning and differentiation depend on Fgf10 signaling [15], whereas the specification of the liver and ventral pancreas is regulated by the transcription factors Prox1 [16], [17] and Pdx1 [18], respectively. In the liver, hepatocytes and intrahepatic biliary cells derive from bipotential hepatoblasts [19]. Multiple genes encoding Jagged ligands and Notch receptors are expressed in the zebrafish liver during intrahepatic biliary duct formation [20]. Perturbation of Jagged-mediated Notch signaling impairs differentiation and morphogenesis of the intrahepatic biliary cells, whereas constitutive Notch activation induces ectopic bile duct formation [20], [21]. These studies support an evolutionarily conserved role for Notch signaling in intrahepatic duct development. Regarding the intrapancreatic ducts, live-imaging analyses of the *Tg(Nkx2.2a(3.5kb):GFP)* line revealed that they derive from cells in the ventral pancreatic bud that migrate towards the pancreatic islet to initiate the formation of a branched network [18], [22]. Molecular mechanisms regulating the development of the intrapancreatic ducts remain poorly understood.

Studies in mouse have shown that the transcription factor SOX9 is expressed in the intrahepatic and intrapancreatic ducts, as well as in the developing HPD system including the common bile duct, gallbladder and hepatopancreatic ampulla [23]–[25]. *Sox9* belongs to the SRY-related box (SOX) gene family that encodes transcription factors containing an HMG box DNA-binding domain. In humans, SOX9 is expressed in the fetal brain, liver, testis and skeletal tissue [26]. Haploinsufficiency of *SOX9* is associated with campomelic dysplasia (CD, OMIM #114290), which is characterized by severe skeletal malformations and sex reversal [26], [27]. More recently, it has been shown that SOX9 is also expressed in the early human fetal pancreas and analysis of CD individuals have revealed pancreatic defects including islet hypoplasia and reduction of hormone expression [28]. Consistent with the defects observed in CD patients, heterozygous knock-out mice are perinatal lethal due to skeletal abnormalities [29]. Conditional knock-out mice have been generated to study SOX9 function: pancreas-specific inactivation of *Sox9* using *Pdx1*:Cre reveals a critical role in the maintenance of the pancreatic progenitor pool [25], whereas liver-specific inactivation of *Sox9* using *Albumin/α-fetoprotein (Alfp)*:Cre shows that it is required for the timely maturation of asymmetrical structures to symmetrical biliary ducts [23]. A potential role for SOX9 in HPD development has not yet been investigated due to the lack of a HPD-specific Cre line.

The zebrafish genome contains two *sox9* orthologs, *sox9a* and *sox9b*, which exhibit partially overlapping expression patterns in the craniofacial cartilage, otic placodes and pectoral appendages [30]. Null mutants of *sox9a* exhibit cartilage defects that mimic those observed in human CD [31]. Although a similar phenotype has been reported for the *sox9b^b971^* mutant [30], the chromosomal deletion which underlies the *b971* lesion removes eleven other genes, greatly limiting the use of this allele to study the function of Sox9b.

Here, we dissect the requirement for Sox9b in the development of the pancreaticobiliary ductal system in zebrafish. We show that similar to mammalian *Sox9*, zebrafish *sox9b* is expressed in the pancreaticobiliary ductal system. Detailed phenotypic analysis of a *sox9b* TILLING mutant reveals that Sox9b regulates the formation of the HPD system as well as the morphogenesis of the intrapancreatic and intrahepatic ducts. Strikingly, the pancreaticobiliary phenotypes observed in larvae worsen during juvenile stages and lead to cholestasis in the homozygous mutant adult fish. We also observed a positive feedback loop between Sox9b and Notch signaling in the developing intrahepatic biliary cells: Notch signaling regulates *sox9b* expression, and in turn Sox9b is required to maintain Notch activity in the intrahepatic biliary cells.

## Results

### *sox9b* is expressed in the developing pancreaticobiliary ductal system

**Figure 1:**
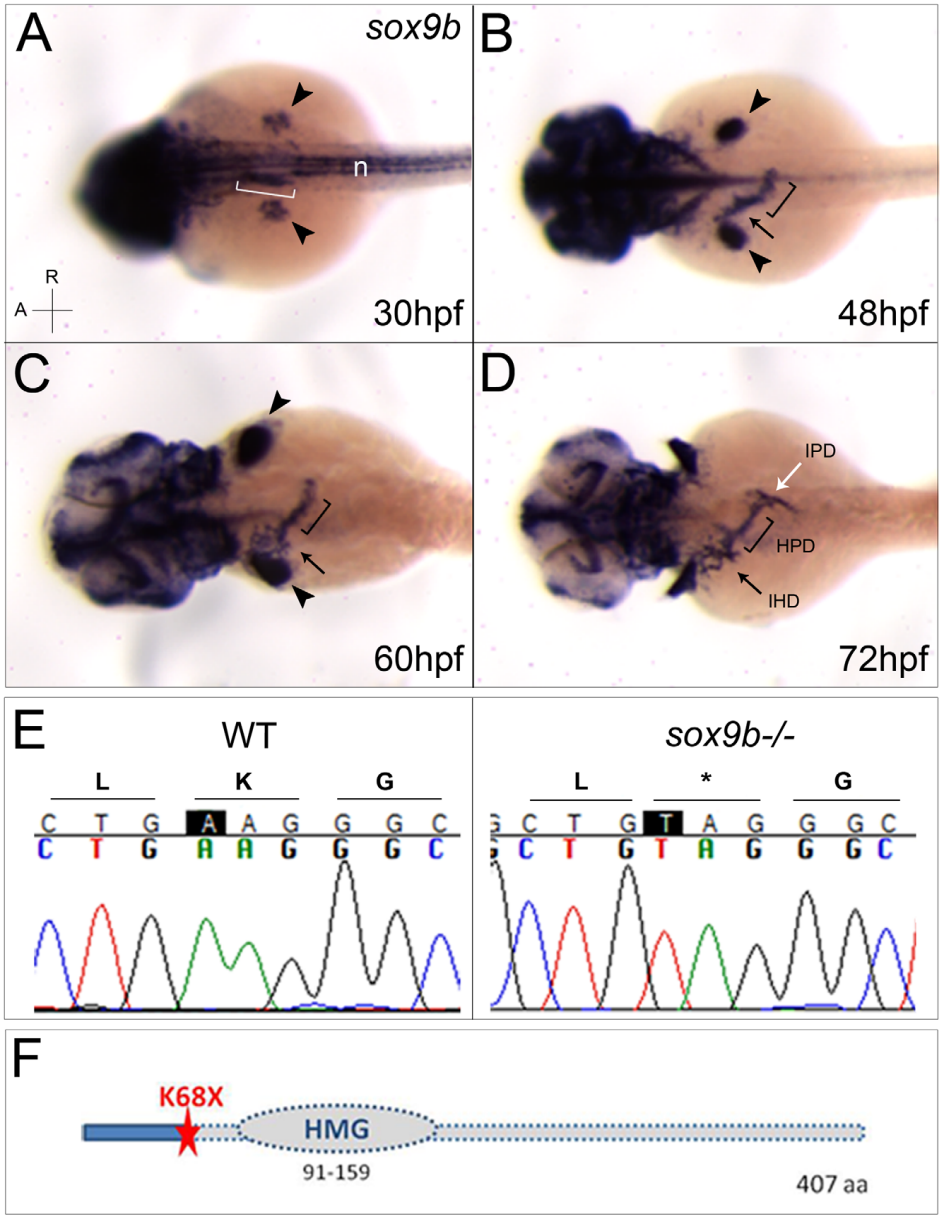
*sox9b* is expressed in the pancreaticobiliary ductal system, and the *sox9b^fh313^* lesion is a nonsense mutation. (A–D) *sox9b* expression is observed in the head and fin buds (arrowheads in A–C), as well as the notochord (n) and part of the foregut endoderm (bracket) at 30 hpf (A). At 48 hpf, *sox9b* expression is observed in the liver bud (arrow) and hepatopancreatic duct primordium (bracket) (B). At 60 hpf, *sox9b* expression covers the intra- (arrow) and extrahepatic (bracket) ducts (C), and extends to the intrapancreatic ducts by 72 hpf (white arrow, D). Dorsal views, anterior (A) to the left. (E) Genomic DNA sequence of wild-type (left panel) and *sox9b^fh313^* mutant (right panel) showing an A>T transversion at position 302 of the coding sequence. This mutation leads to a stop codon at amino acid Lys68. (F) Schema of Sox9b depicting the localization of the amino acid affected by the *fh313* mutation leading to a stop codon upstream of the HMG box DNA-binding domain. IPD, intrapancreatic duct; IHD, intrahepatic duct; HPD, hepatopancreatic duct; HMG, high-mobility group; aa, amino acid.

Intrigued by the recent data revealing *Sox9* expression in the ductal trees of the liver and pancreas as well as in the HPD system in mouse [24], we analyzed the expression pattern of *sox9b* in zebrafish by *in situ* hybridization. We found that in addition to the head region and pectoral fins, *sox9b* is specifically expressed in the pancreaticobiliary ductal system (Figure 1A–1D). At 30 hours post fertilization (hpf), *sox9b* is expressed in a segment of the foregut endoderm (bracket, Figure 1A) that appears to give rise to the liver bud (arrow, Figure 1B) and the HPD primordium (bracket, Figure 1B). At 60 hpf, *sox9b* expression becomes evident in the intrahepatic ducts (arrow, Figure 1C) and then extends to the extra- and intrapancreatic ducts (white arrow, Figure 1D). In contrast to *sox9b*, *sox9a* does not appear to be expressed in the pancreaticobiliary ductal system in zebrafish (Figure S1, left panel). These data show that zebrafish *sox9b* recapitulates the expression pattern of mammalian *Sox9* in the intrapancreatic and intrahepatic ducts as well as in the HPD system [23]–[25].

### *sox9b^fh313^* lesion is a nonsense mutation

To investigate the potential role of Sox9b in the formation of the pancreaticobiliary ductal system, we isolated a novel mutation in *sox9b*, *sox9b^fh313^*, in collaboration with the Zebrafish TILLING Consortium. Contrary to *sox9b^b971^* which consists of a deletion of the lower tip of linkage group 3 [30], *sox9b^fh313^* is a point mutation located in the first exon of *sox9b* (Figure 1E). The A to T transversion at position 302 leads to a premature stop codon at amino acid Lys68. This nonsense mutation likely leads to the synthesis of a truncated protein that lacks the HMG-box DNA binding domain (Figure 1F) and therefore would be non-functional. *In situ* hybridization analyses revealed a substantial decrease of *sox9b* expression in *sox9b^fh313^* mutants at 72 hpf (data not shown), possibly via nonsense mediated mRNA decay. In order to analyze a potential redundancy between *sox9* gene functions in zebrafish, we examined the expression of *sox9a* in *sox9b^fh313^* mutants (Figure S1). As in wild-type, *sox9a* expression appears to be excluded from the digestive organs in *sox9b^fh313^* mutants, suggesting that *sox9a* expression does not compensate for the reduction of Sox9b function in these mutants. Hence, *sox9b^fh313^* is the first point mutation described for this gene in zebrafish and is likely to represent a severe loss-of-function allele.

### *sox9b* mutants survive to adulthood and exhibit cholestasis associated with hepatic and pancreatic fibrosis and duct dilation

**Figure 2:**
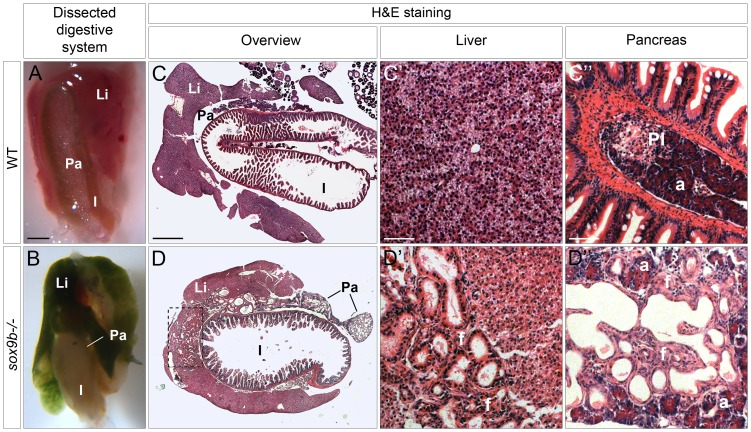
Adult *sox9b* mutants develop cholestasis associated with fibrosis, duct proliferation, and dilation in both the liver and pancreas. (A–B) Dissection of the digestive system of 5 month-old wild-type (A) and *sox9b* homozygous mutant (B) fish reveals a green mutant liver and pancreas reflecting the accumulation of bile in both organs. Anterior to the top. (C–D) Hematoxylin-and-eosin (H&E) staining of histological cross-sections of wild-type (C) and *sox9b* mutant (D) digestive tracts shows abnormal duct morphology in the mutant liver and pancreas. These duct malformations are focalized around the connection with the extrahepatic duct (dashed rectangle in D) in the liver, whereas they are spread over the entire pancreas. Higher magnifications of liver (C′–D′) and pancreas (C″–D″) reveal dilated ducts surrounded by fibrotic tissue (pink staining in D″ labeled as “f”) in both organs in *sox9b* mutants. Li, liver; Pa, pancreas; I, intestine; PI, primary islet; a, acinar compartment. Scale bars, 1 mm in A–B; 500 µm in C–D; 50 µm in C′–D″.

In contrast to a previous report describing the phenotypes of *sox9b^b971^* mutants and *sox9b* morpholino-injected embryos [30], homozygous *sox9b^fh313^* mutants exhibit a normal external morphology and do not show a curly-down body axis or craniofacial defects (data not shown). *sox9b* mutants survive to the adult stage but are much thinner than their wild-type or heterozygous siblings (data not shown). Dissection of the digestive system of 5-month old *sox9b* mutants revealed preserved anatomical relationships, including a three-lobed liver and correctly-looped intestine; however, both the liver and pancreas were strikingly dark green suggesting abnormal bile accumulation (cholestasis) in these organs (Figure 2A–2B). Hematoxylin-and-eosin staining of histological sections of mutant organs showed that both organs exhibited lesions with extensive proliferation and dilation of the ducts, which were surrounded by fibrotic tissue (Figure 2C–2D″). Interestingly, in the liver, ductal defects were restricted to the region that connects to the extrahepatic ductal system (dashed rectangle, Figure 2D) whereas the rest of the organ was much less affected. In contrast, ductal defects in the pancreas were present throughout the organ and worsened towards its distal part (Figure 2D). In the pancreas, the acinar compartment was greatly reduced and secondary islets could not be detected in the sections examined (Figure 2D″).

### Sox9b is required for the patterning and differentiation of the HPD system

**Figure 3:**
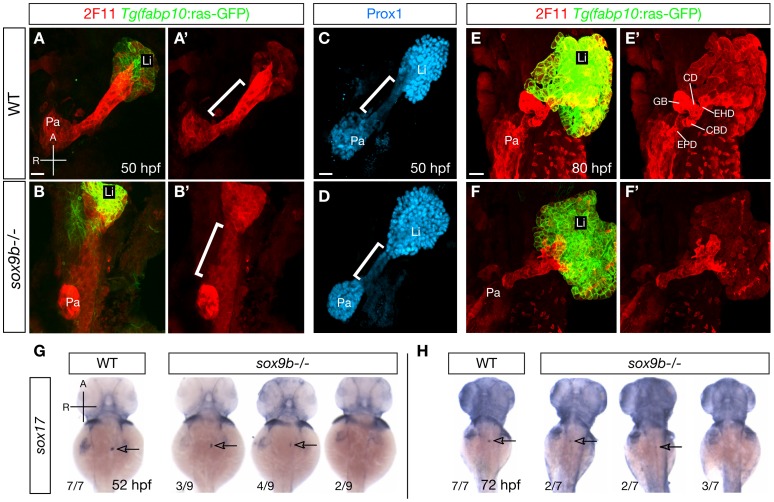
*sox9b* mutants display defective HPD patterning during organ development. (A–B) Labeling for the hepatocyte marker *Tg(fabp10:*ras-GFP) (green) and the HPD marker 2F11 (red) in wild-type and *sox9b* mutant embryos at 50 hpf. 2F11 labeling in the HPD primordium is noticeably reduced in *sox9b* mutants. (A′–B′) Same views as (A–B), but only showing the 2F11 immunostaining. (C–D) In wild-type, Prox1 expression marks the liver and pancreas and is largely absent from the HPD primordium (C). In contrast, Prox1 is abnormally expressed in the HPD primordium in *sox9b* mutants (D). (A′–B′, C–D) Brackets mark the HPD primordium. (E) By 80 hpf, different compartments of the HPD system, including the cystic duct (CD), common bile duct (CBD), gallbladder (GB), extrahepatic duct (EHD) have become evident in wild-type. (F) In *sox9b* mutants, the HPD system is dysmorphic and its compartments are indistinguishable based on morphology. The gallbladder is also often missing. (E′–F′) Same views as (E–F), but only showing 2F11 immunostaining. (G–H) Whole-mount *in situ* hybridization showing *sox17* expression in the gallbladder primordium in wild-type and *sox9b* mutants at 52 (G) and 72 (H) hpf. *sox17* expression is greatly reduced or absent in *sox9b* mutants. The proportion of mutants showing the corresponding phenotype is indicated. Arrows point to the gallbladder primordium. (A–F, A′–F′) All images are projections of confocal z-stacks. Ventral views. (G–H) Dorsal views. Anterior (A) to the top. Pa, pancreas; Li, liver; EPD, extrapancreatic duct. Scale bars, 20 µm.

Due to the robust and highly conserved expression of *sox9b* in the pancreaticobiliary ductal system and the striking liver and pancreas phenotypes seen in the adult mutant fish, we decided to further investigate the roles of Sox9b in the development of these tissues. In zebrafish, the HPD system exhibits unique gene expression profiles that separate it from the liver and pancreas starting at early developmental stages [15]. At 50 hpf, the primordium of the HPD system can be distinguished by strong labeling with the 2F11 antibody, whose antigen remains to be identified [15], [32] (bracket, Figure 3A, 3A′), and low expression of the transcription factor Prox1 [15] (bracket, Figure 3C). In contrast, the liver and pancreas exhibit moderate labeling of 2F11 (Figure 3A, 3A′), but high expression of Prox1 (Figure 3C). In *sox9b* mutants, 2F11 labeling was mostly absent from the region where the presumptive HPD primordium resides (bracket, Figure 3B, 3B′). Moreover, we observed elevated expression of Prox1 in the same region (bracket, Figure 3D). 2F11 labeling showed that by 80 hpf, the HPD system in wild-type larvae has developed into different compartments, including the extrahepatic duct, cystic duct, common bile duct, and gallbladder [15] (Figure 3E, 3E′). At the equivalent stage, the differentiation of the HPD system had partially recovered in *sox9b* mutants as suggested by 2F11 labeling. However, it was severely dysmorphic, with no clear morphological distinction between the cystic duct, extrahepatic duct, and common bile duct (Figure 3F, 3F′). Furthermore, the mutant HPD system often seemed to intrude into the liver (Figure 3F), which was never observed in wild-type larvae. These data indicate that the HPD primordium in *sox9b* mutants exhibits patterning and differentiation defects.

Concordant with the dysmorphic HPD system, the gallbladder in *sox9b* mutants was often indistinguishable based on morphology (Figure 3F and Figure S3A, S3B). We analyzed the expression of *sox17* which marks the gallbladder and its primordium from 36 hpf to 5 days post-fertilization (dpf) [33], and found that it was greatly reduced or absent in *sox9b* mutants at 52 hpf (Figure 3G) and that it did not recover during later development (Figure 3H). This defect in *sox17* expression supports the notion that gallbladder development is severely impaired in *sox9b* mutants.

### *sox9b* mutants fail to form a complex network of ducts in the pancreas

**Figure 4:**
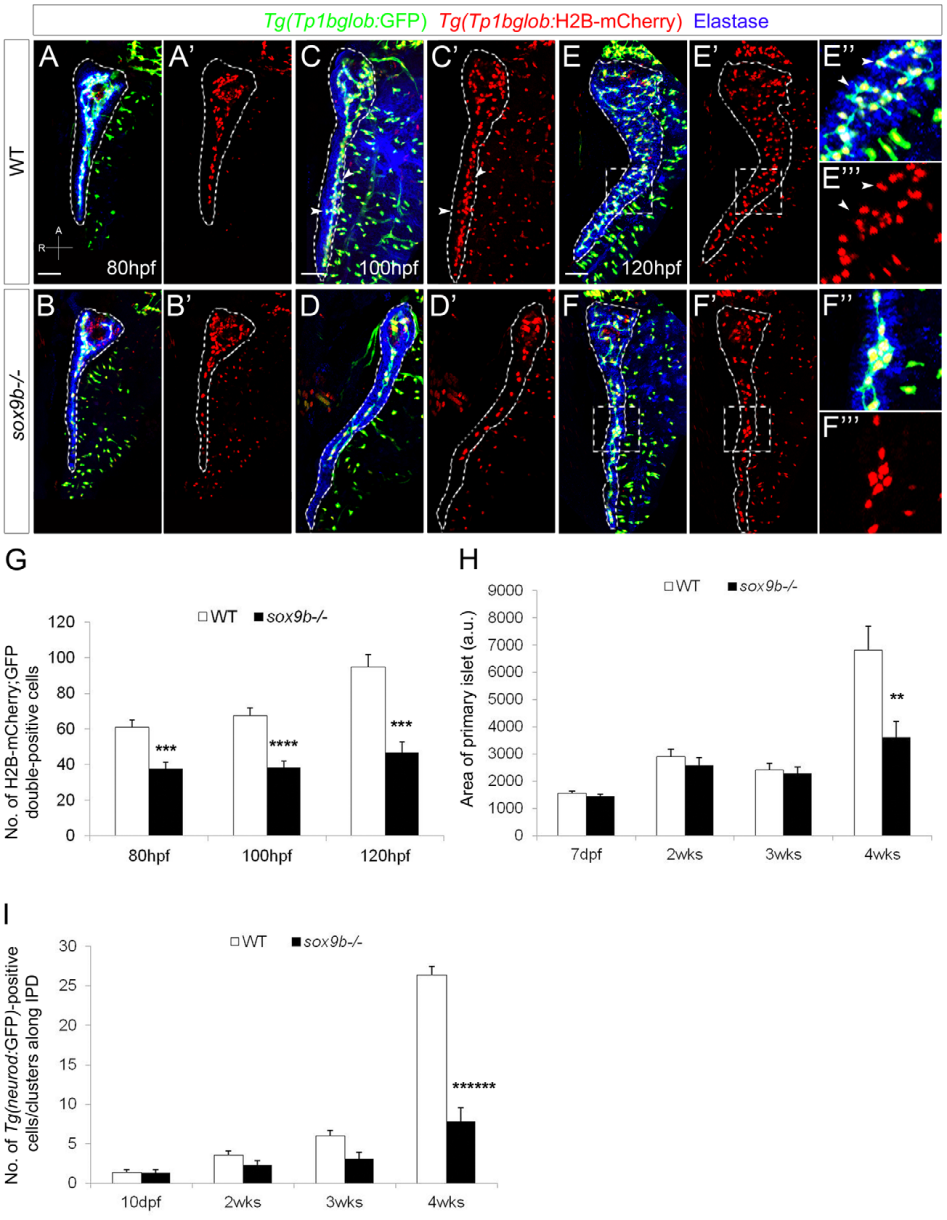
*sox9b* mutant larvae fail to form a complex intrapancreatic ductal network. (A–F) Confocal images of *Tg(Tp1bglob:GFP);Tg(Tp1bglob:H2B-mCherry)* wild-type (top row) and *sox9b* mutant (bottom row) pancreata at 80 (A,B), 100 (C,D) and 120 (E,F) hpf. Elastase antibody staining (blue) labels acinar cells. Although acinar and endocrine tissues appear morphologically unaffected in *sox9b* mutants (data not shown), the intrapancreatic ductal network is less complex and secondary branches are missing in the mutants (D–D′) whereas they start to form by 100 hpf in wild-type larvae (arrowheads and insets, C–C′). (E′″–F′″) Higher magnifications of the area marked by dashed squares in (E–F′) show that at 120 hpf the main duct forms secondary branches (arrowheads) in wild-type larvae (E″–E′″), whereas in the mutants, secondary branches remain absent and clusters of ductal cells are sometimes observed (F″–F′″). (A–F) All images are projections of confocal z-stacks. Ventral views, anterior (A) to the top. Scale bars, 50 µm. (G) Graph representing the number of *Tg(Tp1bglob:*GFP*);Tg(Tp1bglob:*H2B-mCherry)-double positive cells (average±SEM) in the intrapancreatic ducts of wild-type and *sox9b* mutant larvae at different time points. 7 to 11 larvae of each genotype were counted at each stage. (H) Graph representing the area (in arbitrary unit, a.u.) of the primary islet (average±SEM) of *TgBAC(neurod:GFP)* wild-type and *sox9b* mutant larvae (7 dpf) and juvenile animals (2, 3 and 4 weeks (wks)). 6 to 11 animals of each genotype were analyzed at each stage. Area of primary islet was determined using ImageJ. (I) Graph representing the number of *TgBAC(neurod:*GFP)-positive cells/clusters (average±SEM) along the intrapancreatic ducts (IPD) in wild-type and *sox9b* mutant larvae (10 dpf) and juvenile animals. 7 to 11 animals of each genotype were analyzed at each stage. Asterisks indicate statistical significance: *p<0.05; **p<0.01; ***p<0.0005; ****p<0.0001; ******p<0.000005.

We then addressed the role of Sox9b in intrapancreatic duct formation by using the double transgenic line *Tg(Tp1bglob:GFP)*;*Tg(Tp1bglob:H2B-mCherry)* that expresses both GFP and H2B-mCherry under the control of a Notch-responsive element [34], [35]. This line allows the visualization of the shape and nuclei of the intrapancreatic duct cells, as indicated by the overlapping expression of these fluorescent proteins with ductal markers such as E-cadherin and 2F11 [34], [35]. Intrapancreatic ducts derive from cells within the ventral pancreatic bud that migrate towards, and eventually surround, the principal islet at 48 hpf [22]. From 60 hpf, ductal progenitors start to migrate caudally to form a row of cells that give rise to the main intrapancreatic duct [22] (Figure 4A–4A′). The migration of the ductal progenitors did not seem to be impaired in *sox9b* mutants; however, the number of cells within the intrapancreatic ducts was significantly reduced (Figure 4B–4B′, 4G). In wild-type larvae, at 100 hpf, the pancreatic tail keeps elongating, the number of ductal cells has slightly increased (Figure 4C–4C′, 4G) and secondary branches (arrowheads, Figure 4C–4C′) start to form from the main duct. In contrast, in *sox9b* mutants, the number of ductal cells did not increase from 80 to 100 hpf, and no secondary branches appeared, resulting in a primitive ductal system (Figure 4D–4D′, 4G). At 120 hpf, the ductal network in wild-type larvae has become more complex with numerous secondary branches (arrowheads, Figure 4E″) spreading over the acinar compartment (Figure 4E–4E′″). In contrast, the intrapancreatic ductal system in *sox9b* mutants remained poorly developed and clusters of cells could be observed along the main duct (Figure 4F–4F′″), which was still devoid of secondary branches. These data indicate that fewer intrapancreatic duct cells differentiate in the mutants and those that do fail to undergo branching morphogenesis. Furthermore, the number of ductal cells in *sox9b* mutants did not increase as in wild-types. Such a defect is likely due to a problem with cell differentiation as we did not observe any obvious differences in ductal cell proliferation or survival between wild-types and *sox9b* mutants (data not shown).

Given that the adult mutant pancreas exhibits a loss of acinar and potentially endocrine tissues, we analyzed the formation of these compartments during larval and juvenile stages. As assessed by Elastase staining, *sox9b* mutants showed apparently normal proportion of pancreatic acinar tissue at all stages analyzed (Figure 4B, 4D, 4F and Figure S2B″, S2D″, S2F″). As for the endocrine tissue, we investigated the morphology of the primary islet by analyzing *TgBAC(neurod:*GFP) expression which marks early endocrine cells [36]. At late larval (7 dpf) as well as juvenile (2 and 3 weeks) stages, the area of the primary islet appeared similar in wild-type and *sox9b* mutant animals (Figure 4H), suggesting that Sox9b is not required for primary islet formation. At 4 weeks of age, the *sox9b* mutant primary islet was half the size of the wild-type primary islet (Figure 4H). However, it is important to note that at this stage, *sox9b* mutant pancreata were also much less developed than wild-type pancreata (Figure S2E′″, S2F′″). Indeed, *sox9b* juvenile mutants often exhibit growth retardation compared to wild-types and thus, the smaller size of the primary islet could be attributed to an overall growth defect.

In addition to the primary islet, we investigated the formation of secondary islets that arise from progenitors in the intrapancreatic ducts [34], [35] and that, during larval and juvenile stages, appear as small clusters of delaminated cells [34]. We decided to also count single *TgBAC(neurod:*GFP)-positive cells that recently delaminated from the ducts and assumed an endocrine fate. Hence, counting the number of *TgBAC(neurod:*GFP)-positive cells/clusters along the intrapancreatic ducts (Figure S2A′–S2F′), we observed a difference between wild-type and *sox9b* mutant animals at two weeks of age. At 3 and 4 weeks of age, this difference became more pronounced with respectively a 50% and 80% decrease in *TgBAC(neurod:*GFP)-positive cell/cluster number in *sox9b* mutants (Figure 4I). Given that the mutant intrapancreatic ductal network remained primitive and failed to expand at juvenile stages (Figure S2D′″, S2F′″), the defect in secondary islet formation could be related to the lower number of progenitors within the pancreas. Altogether, these data indicate that Sox9b function is required for the development of the intrapancreatic ductal system as well as - directly or indirectly - for the formation of secondary islets.

### Sox9b regulates intrahepatic biliary duct morphogenesis and bile canaliculi formation

**Figure 5:**
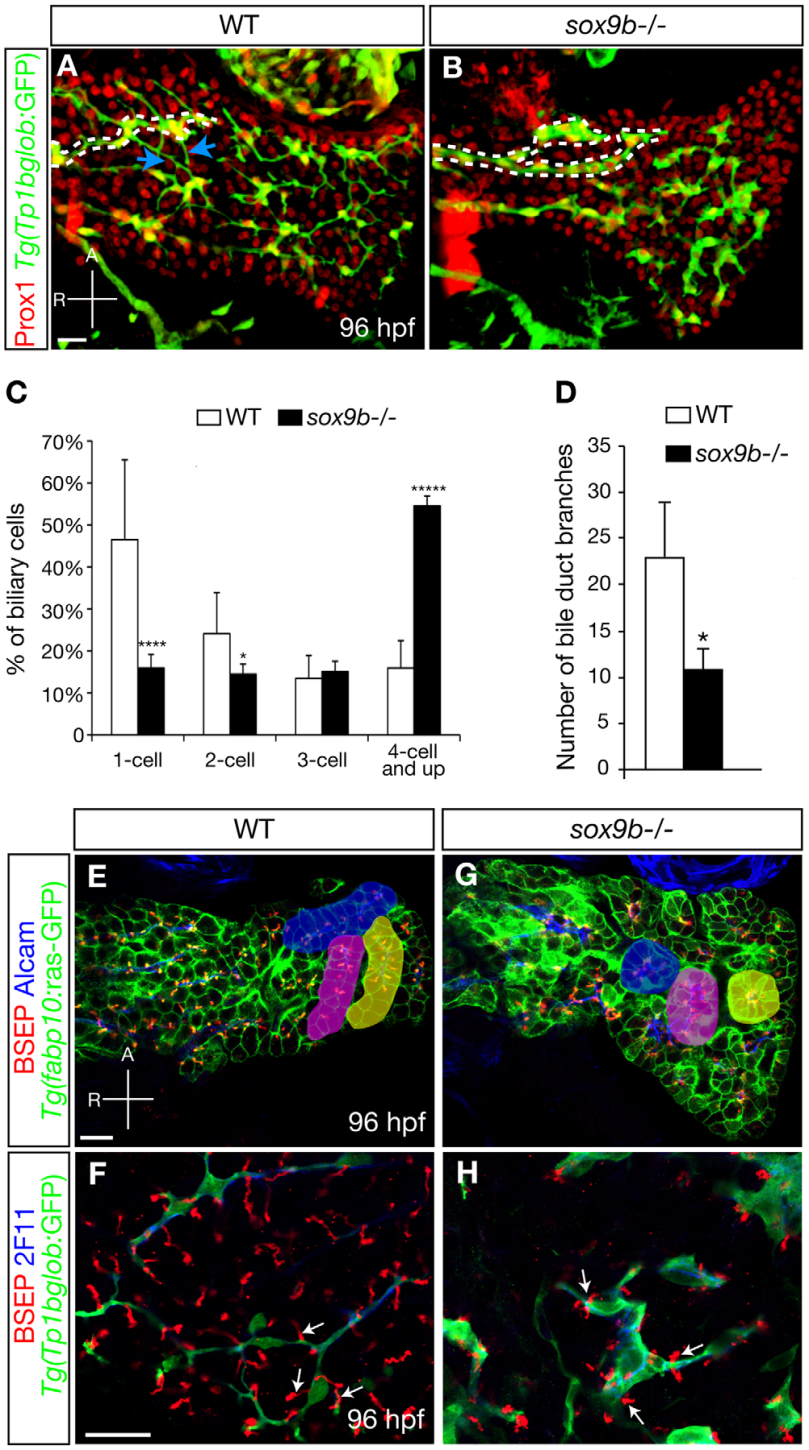
Intrahepatic biliary network morphogenesis and bile canaliculi formation are impaired in *sox9b* mutants. (A–B) Distribution of hepatocytes and biliary cells in 96 hpf wild-type and *sox9b* mutant larvae as revealed by Prox1 (red) and *Tg(Tp1bglob:*GFP) (green) expression. In wild-type, the cell bodies of biliary cells are separated from one another and interconnected via cellular processes (A). In *sox9b* mutants, the biliary cells are clustered together (B). Dashed lines mark the long bile ducts and arrows in (A) point to the short interconnecting ducts that are missing in the mutants. (C) Percentage (average±SEM) of biliary cells that exist as single cells, doubles, triples, or clusters of four or more at 96 hpf. 6 wild-type and 6 *sox9b* mutant larvae were analyzed. (D) Number (average±SEM) of branching points observed along the bile ducts per liver. 7 wild-type and 7 mutants were analyzed. (C–D) Asterisks indicate statistical significance: *p<0.05; ****p<0.0001; *****p<0.00001. (E, G) Wild-type and *sox9b* mutant livers stained for BSEP (red) which marks the canaliculi, Alcam (blue) which marks the apical side of the hepatocytes, and *Tg(fabp10:*ras-GFP) (green) which labels the hepatocytes. The hepatocytes in wild-type livers are organized in parallel arrays (highlighted by pseudo-colors), while the *sox9b* mutant hepatocytes are arranged in rosettes (highlighted by pseudo-colors). (F, H) High magnification confocal images showing the morphology of biliary cells and bile canaliculi. The canaliculi (arrows) in *sox9b* mutant livers appear shorter and wider compared to wild-type. (A–B) All images are projections of confocal z-stacks. (E–H) Single confocal plane images. (A–B, E–H) Ventral views, anterior (A) to the top. Scale bars, 20 µm.

To examine the role of Sox9b in intrahepatic biliary development, we used the *Tg(Tp1bglob:GFP)* line which also marks the intrahepatic biliary cells [21], [35]. During zebrafish liver development, the intrahepatic biliary cells undergo significant morphological changes, whereby these initially contiguous cells separate from one another and interconnect via cytoplasmic processes [21]. By 96 hpf, the wild-type intrahepatic biliary system is composed of a lattice-like network of long ducts joined by short interconnecting ducts [21] (Figure 5A). At the equivalent stage, *sox9b* mutant livers contained similar numbers of intrahepatic biliary cells and hepatocytes as wild-type (data not shown), suggesting that differentiation of the biliary cells is not affected in the mutants. We did not detect any apoptosis of the biliary cells in wild-type or mutant larvae. Strikingly, we observed that most of the biliary cells in the mutant livers failed to separate from one another (Figure 5B). We quantified the percentage of single intrahepatic biliary cells versus cells in cluster of two, three or four and more cells, and found a significant decrease in the percentage of single intrahepatic biliary cells in *sox9b* mutants compared to wild-types concomitant with a significant increase in the percentage of cells in clusters of four and more cells (Figure 5C). Moreover, the long bile ducts in the mutants appeared to be wider than those in wild-types (diameters of the mutant ducts: 3.5 µm or wider; wild-type ducts: 2.5 µm or thinner), and were less branched (Figure 5D).

We then used the *Tg(fabp10:ras-GFP)* line [37] to analyze hepatocyte organization, and co-labeled the animals with an antibody against the bile transporter BSEP to mark the bile canaliculi [38] (Figure 5E, 5G). At 96 hpf, hepatocytes in wild-type livers are arranged as tubules surrounding intrahepatic biliary ducts [20] (Figure 5E). Bile canaliculi are located on the hepatocyte apical membrane which can be marked by the activated leukocyte cell adhesion molecule Alcam [39]. However, in *sox9b* mutants, hepatocytes often formed spherical rosettes with bile canaliculi and Alcam expression located in the center (Figure 5G). This phenotype coincided with the aberrant clustering of intrahepatic biliary cells. Moreover, we found that the canaliculi in *sox9b* mutants appeared to be shorter and wider compared to wild-types (arrows, Figure 5F, 5H), which is consistent with recent data showing the highly coordinated development of intrahepatic biliary cells and bile canaliculi [21].

In liver-specific *Sox9*-inactivated mice, intrahepatic biliary duct morphogenesis is delayed until birth [23], which incited us to track the development of the intrahepatic biliary system in wild-types and *sox9b* mutants during juvenile stages. We found that intrahepatic biliary duct morphogenesis did not recover in *sox9b* mutants and that these animals did not generate morphologically normal bile ducts (Figure S2G–S2L). These data show that, despite a conserved requirement for SOX9 in intrahepatic biliary duct development, zebrafish *sox9b* mutants exhibit a much more severe intrahepatic biliary duct phenotype than the liver-specific knockout mouse model.

### Loss of Sox9b function causes severe defects in bile transport

To determine whether the cholestasis-like phenotype observed in adult *sox9b* mutants occurred during early larval development, we administered fluorescent lipid analogs used to visualize bile transport [40] to 6 dpf-old wild-type and mutant larvae. These fluorescent analogs consist of fatty acids with acyl chains of 5- and 2-carbons (C5:0 and C2:0, respectively) tagged with the BODIPY fluorophore. We selected these two analogs because of the different cells and subcellular details each analog reveals following ingestion. BODIPY-FL C5:0 reveals a high degree of subcellular detail in hepatocytes and acinar cells, such as lipid droplets and zymogen granules, as well as in the ductal networks in the liver and pancreas. The shorter BODIPY-FL C2:0 illuminates the hepatic and pancreatic ducts, as well as the gallbladder providing a functional readout of gallbladder and ductal integrity.

Wild-type larvae fed BODIPY-FL C2:0 exhibited a strong fluorescence signal in their gallbladders (Figure S3A), indicating that bile production, drainage and accumulation was normal. Conversely, no gallbladder BODIPY-FL C2:0 signal was observed in *sox9b* mutants, consistent with their defective gallbladder development (Figure S3B). In the pancreas, BODIPY-FL C2:0 fluorescence was detected throughout the entire intrapancreatic ductal network in wild-type larvae (Figure S3E, S3E′), while it was restricted to the anterior region of the pancreas in *sox9b* mutants (Figure S3F, S3F′), suggesting that the distal intrapancreatic ducts were not functional. Moreover, we observed large pools of fluorescent fluid accumulating in the mutants' pancreatic tail (Figure S3F), which is not typically observed in wild-type larvae unless the gallbladder ruptures. This abnormal extracellular accumulation of fluid (likely pancreatic juice or bile) in and around *sox9b* mutant pancreata is consistent with their malformed pancreaticobiliary ductal system.

Administering BODIPY-FL C5:0 to *sox9b* mutant livers confirmed the dilation and lack of branching morphogenesis of the intrahepatic biliary ducts described above as well as the deformation of bile canaliculi in hepatocytes (Figure S3C, S3C′, S3D, S3D′). Taken together, these data support the notion that the cholestasis-like phenotype observed in the adult *sox9b* mutants results from defects in early ductal morphogenesis.

### Notch regulates *sox9b* expression during intrahepatic biliary network formation

**Figure 6:**
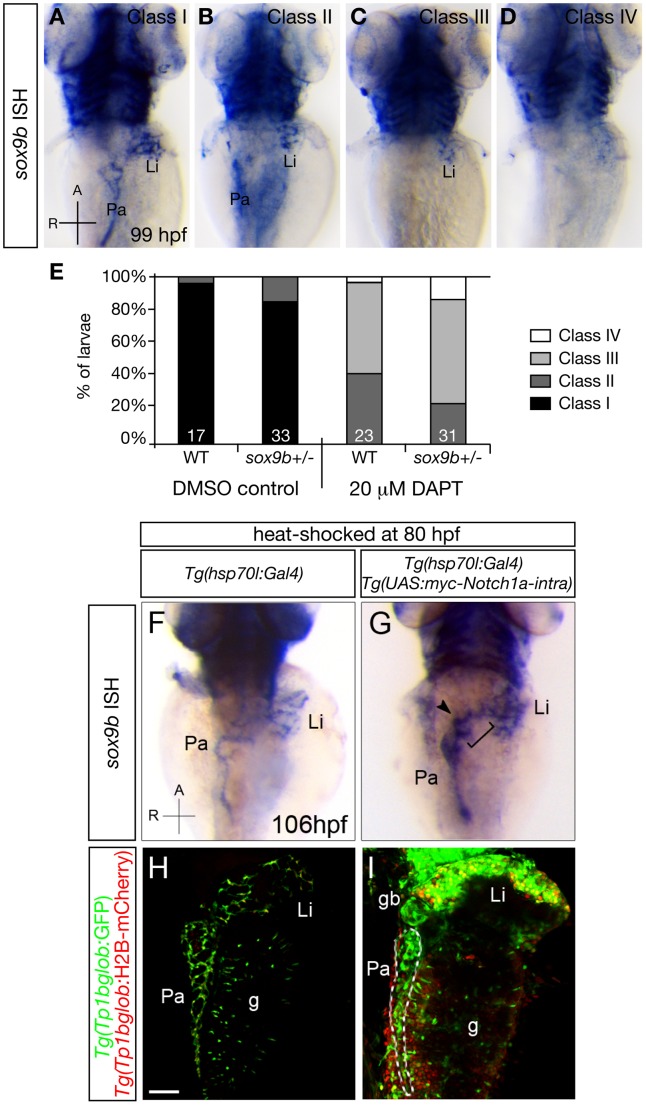
Notch signaling regulates *sox9b* expression in the intrahepatic and intrapancreatic ducts. (A–D) Whole-mount *in situ* hybridization (ISH) showing *sox9b* expression in larvae obtained from a wild-type to *sox9b* heterozygote cross that were treated with DMSO control or 20 µM DAPT between 75 and 99 hpf. In DMSO-treated controls, *sox9b* was strongly expressed in the liver and pancreas at 99 hpf (A, Class I). In animals treated with 20 µM DAPT (B–D), some showed a slight reduction of *sox9b* expression in the liver (Li) and pancreas (Pa) (B, Class II), others only retained expression of *sox9b* in the liver (C, Class III), and the remaining ones did not exhibit any obvious expression of *sox9b* in either organ (D, Class IV). Dorsal views, anterior (A) to the top. (E) Percentages of larvae showing different classes of phenotypes. *sox9b* heterozygotes exhibited a more severe reduction in *sox9b* expression than wild-type upon DAPT treatment. The numbers of larvae analyzed are indicated at the bottom. (F–I) Larvae obtained from crossing *Tg(hsp70l:Gal4)*;*Tg(UAS:myc-Notch1a-intra)* hemizygous and *Tg(Tp1bglob:GFP);Tg(Tp1bglob:H2BmCherry)* parents were heat-shocked at 80 hpf to induce myc-Notch1a-intra expression and fixed 26 hours later. (myc-Notch1a-intra)-overexpressing larvae were selected based on the increased expression of both *Tg(Tp1bglob:*GFP) and *Tg(Tp1bglob:*H2BmCherry), and their genotype was confirmed by anti-myc antibody labeling (data not shown). (F–G) *in situ* hybridization showed an increased expression of *sox9b* in the liver (Li) and pancreas (Pa) as well as in the HPD system (bracket) including the gallbladder (arrowhead) in (myc-Notch1a-intra)-overexpressing larvae (G) compared to (myc-Notch1a-intra)-negative siblings (F). 10 larvae of each genotype were analyzed. Dorsal views, anterior (A) to the top. (H–I) Confocal images of (myc-Notch1a-intra)-negative (H) and (myc-Notch1a-intra)-overexpressing (I) larvae showing elevated expression of *Tg(Tp1bglob:*GFP) and *Tg(Tp1bglob:*H2BmCherry) in the liver (Li), pancreas (Pa), gut (g) and gallbladder (gb) in (myc-Notch1a-intra)-overexpressing larvae after heat-shock. Dashed lines in (I) mark the pancreas. All images are projections of confocal z-stacks. Ventral views, anterior (A) to the top. Scale bars, 50 µm.

The defects in intrahepatic biliary ducts and bile canaliculi observed in *sox9b* mutants are strikingly similar to those reported in the mouse and zebrafish models of Notch deficiency [6], [9], [20], [21], [41]. In particular, it has been shown in zebrafish that Notch signaling directs the segregation of intrahepatic biliary cells between 70 and 96 hpf [21]. Given that the intrahepatic biliary cells in *sox9b* mutant livers fail to separate from one another, we hypothesized that Sox9b interacts with Notch signaling to regulate the morphogenesis of the intrahepatic biliary ducts. To test whether inhibiting Notch signaling affects *sox9b* expression, we treated wild-type and *sox9b* heterozygous animals with a low dose of the γ-secretase inhibitor DAPT from 75 to 99 hpf [42], and assessed *sox9b* expression by *in situ* hybridization (Figure 6A–6E). Such DAPT treatment caused a reduction in *sox9b* expression which was more pronounced in *sox9b* heterozygotes than in wild-types. We also performed the reverse experiment by using *Tg(hsp70l:Gal4);Tg(UAS:myc-Notch1a-intra)* hemizygous larvae to induce ubiquitous overexpression of the Notch intracellular domain (NICD) upon heat-shock treatment [43]. These animals were heat-shocked at 80 hpf and *sox9b* expression was analyzed 26 hours later by *in situ* hybridization. The heat-shock treatment efficiently induced overactivation of Notch signaling activity in the *Tg(Tp1bglob:GFP); Tg(Tp1bglob:H2B-mCherry)* larvae (Figure 6H, 6I). We observed an increase in *sox9b* expression throughout the pancreas, liver and HPD system (bracket) in the double–transgenic larvae compared to their single-transgenic control siblings (Figure 6F, 6G). Expression of *sox9b* could even be detected in the gallbladder (arrowhead, Figure 6G), which was not seen in control larvae (Figure 6F). This increase in *sox9b* expression is unlikely due to an NICD-induced proliferation of *sox9b*-positive cells because we could already detect higher levels of *sox9b* expression as early as four hours after heat-shock treatment (data not shown). Taken together, these loss- and gain-of-function analyses reveal that Notch signaling regulates *sox9b* expression during intrahepatic biliary duct morphogenesis. These data are consistent with studies in mouse showing that Notch1 can directly bind to the *Sox9* promoter [9].

### Sox9b is required to maintain Notch signaling in the intrahepatic biliary duct cells

To further analyze the dynamics of Notch signaling in *sox9b* mutants, we utilized *Tg(Tp1bglob:H2B-mCherry);Tg(Tp1bglob:VenusPest)* animals in which the Notch-responsive element drives the expression of both H2B-mCherry and VenusPest fluorescent proteins. Contrary to H2B-mCherry which is very stable, the destabilized fluorescent protein VenusPest has a short half-life (2 hours for GFP-Pest in mammalian cells) [44]. Thus, the *Tg(Tp1bglob:*H2B-mCherry);*Tg(Tp1bglob:*VenusPest)-double positive cells are currently Notch responsive, whereas the *Tg(Tp1bglob:*H2B-mCherry)-positive;*Tg(Tp1bglob:*VenusPest)-negative cells were positive for Notch signaling in their recent past but have since switched it off [34].

**Figure 7:**
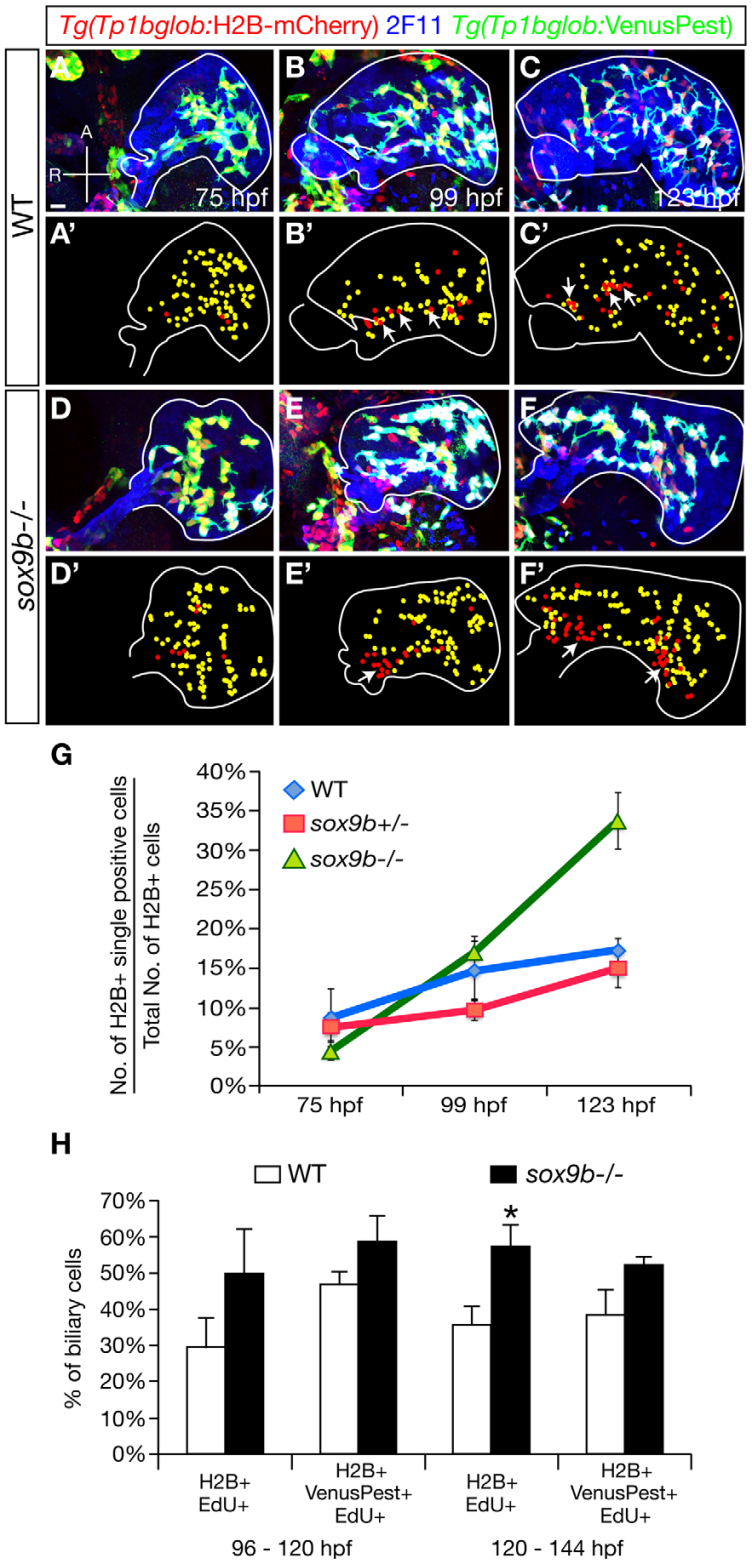
The pattern of Notch signaling activity is altered in *sox9b* mutants. (A–F) Expression of *Tg(Tp1bglob*:H2B-mCherry) (red), 2F11 (blue), and *Tg(Tp1bglob:*VenusPest) (green) in wild-type (A–C) and *sox9b* mutant (D–F) larvae at 75 (A, D), 99 (B, E), and 123 hpf (C, F). At each time point, 6 larvae of each genotype were analyzed. (A′–F′) Diagrams showing the distribution of *Tg(Tp1bglob:*H2B-mCherry);*Tg(Tp1bglob:*VenusPest)-double positive cells (yellow) and *Tg(Tp1bglob:*H2B-mCherry)-single positive cells (red) in (A–F). Livers are outlined by solid white line. In wild-type livers, expression of *Tg(Tp1bglob:*H2B-mCherry) and *Tg(Tp1bglob:*VenusPest) largely overlaps at 75 hpf (A, A′). At 99 and 123 hpf (B–B′, C–C′), a few *Tg(Tp1bglob:*H2B-mCherry)-single positive cells (arrows) appear along the intrahepatic duct that connects to the extrahepatic system. In *sox9b* mutants, *Tg(Tp1bglob:*H2B-mCherry);*Tg(Tp1bglob:*VenusPest)-double positive cells and *Tg(Tp1bglob:*H2B-mCherry)-single positive cells show similar distribution as in wild-type at 75 and 99 hpf (D–D′, E–E′). However, at 123 hpf (F, F′), we observed big clusters of *Tg(Tp1bglob:*H2B-mCherry)-single positive cells in the mutant livers (arrows). (G) Percentages (average±SEM) of *Tg(Tp1bglob:*H2B-mCherry)-single positive cells relative to the total number of *Tg(Tp1bglob:*H2B-mCherry)-expressing cells. Whereas wild-type and *sox9b* heterozygous livers contained similar percentages of *Tg(Tp1bglob:*H2B-mCherry)-single positive cells at all stages examined (p>0.4), this percentage was significantly higher in *sox9b* mutants at 123 hpf (p<0.0005). (H) Percentages (average±SEM) of *Tg(Tp1bglob:*H2B-mCherry)-single positive cells or *Tg(Tp1bglob:*H2B-mCherry);*Tg(Tp1bglob:*VenusPest)-double positive cells that were labeled by EdU. EdU incubation was conducted from 96 to 120 hpf or from 120 to 148 hpf. Under both conditions, the hepatic Notch responsive cells in *sox9b* mutants showed higher EdU incorporation compared to wild-type, and the difference was more pronounced for *Tg(Tp1bglob:*H2B-mCherry)-single positive cells. 7 wild-types and 7 *sox9b* mutants were examined for each experimental condition. Asterisks indicate statistical significance: *, p<0.05. (A–F) All images are projections of confocal z-stacks. Ventral views, anterior (A) to the top. Scale bar, 20 µm.

In wild-type and *sox9b* heterozygous livers, the expression of *Tg(Tp1bglob:*H2B-mCherry) and *Tg(Tp1bglob:*VenusPest) largely overlapped at 75 hpf (Figure 7A, 7A′, 7G, and data not shown). Between 99 and 123 hpf, a small proportion of intrahepatic biliary cells switched off Notch signaling and became *Tg(Tp1bglog:*H2B-mCherry)-single positive (Figure 7B–7C′, G). Up to 99 hpf, *sox9b* mutant livers exhibited a similar pattern of Notch signaling activity as wild-type with a clear overlap between *Tg(Tp1bglob:*H2B-mCherry) and *Tg(Tp1bglob:*VenusPest) expression (Figure 7D–7E′, 7G). However, at 123 hpf, the proportion of *Tg(Tp1bglog:*H2B-mCherry)-single positive cells was significantly higher in *sox9b* mutants compared to wild-types or heterozygotes (Figure 7F, 7F′, 7G), suggesting that *sox9b* mutants fail to maintain Notch signaling in the intrahepatic biliary cells. Interestingly, we did not observe any obvious phenotype when assessing Notch signaling activity in the mutant intrapancreatic ducts.

To follow up this observation, we treated wild-type and mutant larvae with 50 µM DAPT, a dose that only partially inhibits Notch signaling. Upon treatment between 106 and 154 hpf, DAPT-treated wild-type larvae showed a specific loss of Notch signaling activity in the proximal (p) rather than the distal (d) region of the liver (Figure S4C). In DAPT-treated mutant larvae, we observed a drastic loss of Notch signaling throughout the entire liver (Figure S4D, S4E), indicating that *sox9b* mutant biliary cells are more likely to lose Notch signaling activity than wild-type cells. Altogether, these data indicate that Sox9b is required for the maintenance but not the initiation of Notch signaling in the intrahepatic biliary cells. Considering our previous data showing that Notch signaling regulates *sox9b* expression, we hypothesize that Notch and Sox9b interact in a positive feedback loop to ensure the development of the intrahepatic biliary network.

To better understand the biological significance of Notch responsiveness during intrahepatic biliary duct morphogenesis, we compared the distribution of *Tg(Tp1bglob:*H2B-mCherry)-single positive cells and *Tg(Tp1bglob:*H2B-mCherry);*Tg(Tp1bglob:*VenusPest)-double positive cells in wild-type livers. At 123 hpf, 89% of the double-positive cells existed as individual cells connecting to one another through cellular extensions (Figure 7C′; 467 cells in 5 larvae were analyzed). On the other hand, 60% of the *Tg(Tp1bglob:*H2B-mCherry)-single positive cells, which had switched off Notch signaling, were intermingled with each other to form larger groups (Figure 7C′, arrows; 110 cells in 5 larvae were analyzed). Interestingly, these clusters of *Tg(Tp1bglob:*H2B-mCherry)-single positive cells were mostly present in the multicellular large bile ducts contiguous with the extrahepatic duct, whereas the individual *Tg(Tp1bglob:*H2B-mCherry);*Tg(Tp1bglob:*VenusPest)-double positive cells were localized in the distal part of the liver and formed smaller bile ducts (Figure 7C′). These data suggest that Notch responsiveness correlates with the relative position of the intrahepatic biliary cells, with the cells turning off Notch signaling forming the large bile ducts in the proximal region of the liver. In *sox9b* mutant livers, we observed more clusters of *Tg(Tp1bglob:*H2B-mCherry)-single positive cells in both the proximal and distal regions (Figure 7F′, arrows; 243 cells in 6 larvae were analyzed).

We then addressed whether Notch responsiveness was related to the proliferation status of the intrahepatic biliary cells, which would correlate with the increase in ductal structures observed in mutant adults. We incubated wild-type and mutant animals with the replication marker 5-ethynyl-2′-deoxyuridine (EdU) during two intervals of larval development, and analyzed EdU incorporation in *Tg(Tp1bglob:*H2B-mCherry)-single positive and *Tg(Tp1bglob:*H2B-mCherry);*Tg(Tp1bglob:*VenusPest)-double positive cells (Figure 7H). In wild-type larvae, approximately 30% of the *Tg(Tp1bglob:*H2B-mCherry)-single positive cells incorporated EdU after incubation from 96 to 120 hpf. The *Tg(Tp1bglob:*H2B-mCherry);*Tg(Tp1bglob:*VenusPest)-double positive cells exhibited a slightly higher percentage of EdU incorporation, although the difference between these two cell populations was not statistically significant (p>0.08). Similar rates of EdU incorporation were observed when we incubated the animals from 120 to 144 hpf. In *sox9b* mutants, we detected an increase in EdU incorporation in both the single and double positive cells compared to wild-type (Figure 7H), with the increase being more pronounced in the *Tg(Tp1bglob:*H2B-mCherry)-single positive cells than in the double positive cells. To further support the hypothesis that loss of Notch signaling correlates with increased proliferation of biliary cells, we found that partial inhibition of Notch signaling in wild-type larvae by a low dose DAPT treatment led to an increase in biliary cell proliferation similar to that observed in *sox9b* mutants (data not shown). Hence, taken together, these data suggest that the reduction in Notch signaling in *sox9b* mutants promotes the clustering and proliferation of the intrahepatic biliary cells, which is consistent with the biliary duct defects observed in the mutant adults.

## Discussion

In this study, we analyzed a novel *sox9b* mutant in zebrafish, revealing for the first time that global loss-of-function of Sox9b severely impairs the development of the pancreaticobiliary ductal system. In particular, we showed that in the mutant animals, the HPD system is malformed, and the intrahepatic and intrapancreatic ducts fail to form a functional ductal network. We also uncovered the existence of a Notch-Sox9b positive feedback loop that is crucial for intrahepatic biliary duct development. Our study thus brings new insights into our understanding of pancreaticobiliary ductal system formation, an important but understudied process.

We showed that loss of Sox9b function leads to mispatterning of the HPD primordium. Fgf10 signaling also plays a pivotal role in the formation of the HPD system in zebrafish [15]. Zebrafish *fgf10* is expressed in the mesenchyme surrounding the HPD system and intestine but not that surrounding the liver or pancreas [17]. *fgf10* mutants show a dysmorphic HPD with a reduction or loss of the common bile duct as well as reduced extrapancreatic and extrahepatic ducts. Moreover, *fgf10* mutants misexpress hepatic markers such as Prox1 and Hnf4α in their HPD system and pancreas, leading to the ectopic differentiation of some cells in these organs towards a liver fate. Based on the similarities of the *fgf10* and *sox9b* mutant phenotypes, it is possible that Fgf10 and Sox9b interact; for example, Fgf10 signaling could induce or maintain *sox9b* expression in the HPD primordium to modulate the patterning of this tissue. However, we did not observe any obvious change in *sox9b* expression in the HPD upon Fgf receptor pharmacological inhibition treatment, nor in *fgf10* expression in the surrounding mesenchyme in *sox9b* mutants. Thus, Fgf10 and Sox9b functions might intersect in other ways.

Our data show that Sox9b is involved in gallbladder development, whose primordium specifically expresses *sox1*7. s*ox17* is expressed in all endodermal cells during gastrulation [45] and starts to be reexpressed at 36 hpf in a small region of the liver close to the extrahepatic duct [33]. It is then detected in the gallbladder at 60 hpf where it persists until 5 dpf. It will be interesting to determine whether Sox9b directly regulates *sox17* expression and also to identify the additional factors involved in inducing *sox17* expression in a subset of *sox9b* positive cells.

The intrapancreatic ductal network is severely disrupted in *sox9b* mutant larvae, leading to the formation of dilated ducts surrounded by fibrotic tissue in mutant adults. At first glance, this adult phenotype is reminiscent of the one caused by pancreas-specific inactivation of mouse *Sox9* [25]; however, given that the pancreatic remnants described in the mouse mutants come from unrecombined Sox9+ progenitor cells, the zebrafish adult phenotype appears much less severe than the one seen in mouse. The discrepancy between the two models could be explained by differences during early pancreas development. Indeed, mouse SOX9 is expressed in pluripotent pancreatic progenitors and is required to stimulate their proliferation and survival [25]. However, in zebrafish at the earliest stages of pancreas development, endocrine cells derive first from the endodermal epithelium [18] and then from the extrapancreatic duct [46]; only during larval stages, do endocrine cells derive from progenitors within the intrapancreatic ducts [34], likely following mechanisms similar to those regulating the secondary transition in mouse. Analysis of zebrafish *sox9b* expression at early stages suggests that it is present in a subset of ventral pancreatic bud cells, which may correspond to the ductal progenitors. If this interpretation is correct, the lack of Sox9b function in zebrafish would first impair these ductal progenitors, leading to a reduced number of intrapancreatic ductal cells. The first two waves of endocrine cells, as well as the acinar cells, which originate from *sox9b* negative tissue, would therefore not be affected in a *sox9b* mutant.

Loss of Sox9b function in zebrafish severely impairs the development of the intrahepatic biliary network including the morphogenesis of the bile canaliculi. These phenotypes are more severe than those seen in liver-specific SOX9-depleted mice [23]. During mouse liver development, SOX9 expression is first detected at E10.5 in the endodermal cells lining the lumen of the liver diverticulum [23]. This expression is lost as the liver cells migrate into the septum transversum, but re-emerges at E11.5 in cells that form the ductal plate. The *Alfp:*Cre line that was used to recombine the *Sox9* locus becomes active at E11.5, thus likely only inhibiting the second phase of SOX9 expression. Therefore, the phenotypic differences between the zebrafish *sox9b* mutant and the mouse model might be related to the consequences of an earlier depletion in Sox9b in zebrafish than in mouse, illustrating the value of the zebrafish Sox9b global loss-of-function model to uncover functions of this critical transcriptional regulator. However, differences in the expression or function between *sox9b* (zebrafish) and *Sox9* (mouse) may also explain the phenotypic differences between the two models.

Intrahepatic biliary cells in *sox9b* mutant livers fail to segregate from one another and remain clustered, leading to a primitive ductal network. Such defects are strikingly similar to Notch-deficient zebrafish and mouse models [6], [9], [20], [21], [41] which phenocopy the human Alagille syndrome (OMIM#118450), which itself is associated with *JAGGED1* and *NOTCH2* mutations. Notably, this developmental disorder is characterized by cholestasis due to a paucity of biliary ducts. Our data indicate that Sox9b interacts with Notch signaling in a positive feedback loop to regulate intrahepatic biliary duct morphogenesis. Studies in mouse have provided possible mechanisms underlying this Sox9-Notch crosstalk: the *Sox9* promoter displays ten consensus Rbpj binding sites and is a direct target of Notch signaling [9]. In addition, SOX9 modulates Notch signaling by positively regulating the expression of the Notch downstream target gene *Hes1* in the liver [23] as well as in other organs such as the pancreas [25]. These mechanisms are likely to be at play in zebrafish as well, and it will be interesting to delve deeper into the complexities of this positive feedback loop.

Intriguingly, we found that the biliary cells in the proximal region of wild-type livers tend to lose Notch signaling more quickly and are more susceptible to Notch inhibition than the distal cells. Notably, the biliary cells in the proximal region form large ducts whereas the distal cells form small ducts. In *sox9b* mutants, loss of Sox9b function tempers Notch signaling in all biliary cells. Consequently, the mutant livers exhibit aberrant clusters of *Tg(Tp1bglob:*H2B-mCherry)-single positive cells in their distal region, suggesting that lack of Sox9b-mediated maintenance of Notch signaling could promote the formation of large ectopic ducts in distal regions of the liver. Interestingly, it has been shown in mouse that *SOX9*, which is expressed in all biliary cells at E18.5, persists in small ducts but regresses from large ducts after birth [23]. Therefore, it is possible that in wild-type animals, the loss of Notch signaling in the proximal region of the liver induces the local loss of *sox9b* expression, leading to the formation of large bile ducts. To begin to test this hypothesis, it will be necessary to generate a Sox9b antibody in order to examine Sox9b expression at cellular resolution. The identification of markers that distinguish large and small bile ducts would also greatly facilitate such studies.

Our data suggest that in addition to regulating biliary morphogenesis, the Notch-Sox9b module also influences the proliferation of biliary cells. In *sox9b* mutants, the biliary cells that turn off Notch signaling exhibit a higher proliferation rate. Recent lineage tracing studies in mouse have shown that *Sox9* is expressed in liver progenitor cells that reside within the biliary ducts [47], [48]. It will be interesting to determine whether zebrafish *sox9b* is also expressed in liver progenitor cells, whether loss of Sox9b function affects their proliferation rate, and investigate into the underlying mechanisms.

In addition to bringing new insights into our understanding of the development of the pancreaticobiliary ductal system, the analysis of zebrafish *sox9b* mutants should lead one to consider *SOX9* as a candidate gene for human diseases associated with HPD, intrapancreatic or intrahepatic duct malformations. In particular, numerous cases of congenital non-syndromic or syndromic extrahepatic biliary atresia have been reported [49] and their causes remain unknown [50]. These conditions likely have multifactorial causes and do not display simple Mendelian inheritance. Given the expression pattern of SOX9 in mammals [24], [26], [28] as well as the phenotypes caused by loss of SOX9 function in zebrafish and mouse, *SOX9* is therefore an interesting gene to sequence in those patients. Campomelic dysplasia has been shown to be essentially associated with heterozygous mutations that are predicted to severely disrupt SOX9 protein structure and function [51]; but milder lesions could be associated with pancreaticobiliary duct malformations and contribute to the onset or severity of these malformations without necessarily impairing skeletal development.

## Materials and Methods

### Zebrafish strains and lines

Embryos and adult fish were raised and maintained under standard laboratory conditions [52]. The *sox9b^fh313^* heterozygote was crossed with *Tg(fabp10:ras-GFP)^s942^* [37], *Tg(Tp1bglob:GFP)^um14^* [35], *Tg(Tp1bglob:H2B-mCherry)^s939^*, *Tg(Tp1bglob:VenusPest)^s940^* [34], *TgBAC(neurod:GFP)^nl1^* [53] and genotyped according to the TILLING center protocol with AcuI or SfcI (http://labs.fhcrc.org/moens/Tilling_Mutants/sox9b/allele_1.html). We also used *Tg(hsp70l:Gal4)^1.5kca4^* and *Tg(UAS:myc-Notch1a-intra)^kca3^* [43] hemizygous or double hemizygous fish.

### *In situ* hybridization and immunohistochemistry

Whole-mount *in situ* hybridizations were performed as described previously [54] using *sox9b* [30] and *sox17* [45] probes. Animals were photographed with a Zeiss Axioplan using an Axiocam digital camera. Immunohistochemistry on whole-mount animals or cryosections was performed as previously described [16], using the following antibodies: chicken polyclonal anti-GFP (1∶1000; Aves Labs, Tigard, OR, USA), rabbit polyclonal anti-Prox1 (1∶1000; Chemicon, Billerica, MA, USA), mouse monoclonal 2F11 (1∶1000; Abcam, Cambridge, UK), rabbit polyclonal anti-dsRed (1∶500; Clontech, Mountain View, CA, USA), rabbit polyclonal anti-ABCB11/BSEP (1∶1000; Kamiya Biomedical), mouse monoclonal anti-Alcam/Zn8 (1∶20; ZIRC), rabbit polyclonal anti-elastase (1∶200; Millipore AB1216) and fluorescently conjugated Alexa antibodies (1∶250; Molecular Probes, Carlsbad, CA, USA). Samples were imaged on a Zeiss Pascal confocal microscope. The width of the bile ducts was measured using the “local thickness” function in Fiji software.

### Heat-shock experiment and chemical inhibitor treatment

Heat-shock treatments of *Tg(hsp70l:Gal4)^1.5kca4^* larvae were performed at 38°C as described [16]. To inhibit Notch signaling, larvae were treated with 20 µM or 50 µM DAPT (Sigma) in egg water [42]. Control larvae from the same batch were treated with 0.4% DMSO in egg water. Statistical analyses were performed using the Student's two-tailed *t*-test.

### Histology

Five-months old zebrafish (one wild-type and one mutant) were euthanized and their digestive systems were dissected and fixed overnight with formalin at 4°C. The samples were embedded in paraffin, cut into 5 µm sections, and stained with hematoxylin and eosin.

### BODIPY assay

6–7 dpf larvae were fed with BODIPY C2.0 or BODIPY C5.0 as described in [40] for 6–8 hours before being mounted and imaged live. The larvae were subsequently genotyped and 9 wild-type and 9 *sox9b* mutant animals were analyzed.

### EdU cell cycle analysis

To assess the proliferation of the intrahepatic biliary cells, wild-type and *sox9b* mutant larvae were incubated in 7 µM EdU dissolved in egg water during the stages indicated. Control larvae collected from the same batch were treated with 1.7% DMSO. Animals were fixed after incubation and processed using the Click-iT EdU Imaging Kit (Invitrogen). Quantification of EdU incorporation was conducted using the Cell Counter plug-in in ImageJ.

## Supporting Information

Figure S1 *sox9a in situ* hybridization in wild-type and *sox9b* mutant larvae at 80 hpf. *sox9a* does not appear to be expressed in endodermal tissues in wild-type or *sox9b* mutant animals. Dorsal views, anterior (A) to the left.
(TIF)

Figure S2 Time course analyses of the pancreas (A–F) and liver (G–L) in wild-type and *sox9b* mutant larvae at 2, 3, and 4 weeks. (A–F) *Tg(Tp1bglob:H2B-mCherry);TgBAC(neurod:GFP)* double transgenic fish from *sox9b* heterozygote incrosses were raised together and 40 of them were fixed and genotyped at each time point. The pancreas, along with the gut, of 6 to 11 wild-type or *sox9b* homozygous mutant fish were dissected, stained with anti-dsRed (red), anti-GFP (green) and anti-Elastase (blue) antibodies and mounted for confocal imaging. Whereas wild-type pancreata showed complex intrapancreatic duct networks at 2 and 3 weeks of age with several main pancreatic ducts (A, C), *sox9b* mutant pancreata failed to expand and ductal cells stayed in clusters (B, D). At 4 weeks of age, the morphological differences between wild-type and *sox9b* mutant pancreata were even more obvious: wild-type pancreata spread over the gut starting to form lobes whereas *sox9b* mutant pancreata were still primitive in appearance (E, F). The defect in pancreatic growth in *sox9b* mutant fish is associated with a global growth retardation of the fish as indicated by the smaller size of the fish and the frequent occurrence of an unlooped gut at 4 weeks (data not shown). In addition to pancreatic duct morphological defects, *sox9b* mutant fish showed a deficiency in secondary islet formation as assessed by *TgBAC(neurod:*GFP) expression (arrowheads in B′, D′, F′). However, at the equivalent stages, wild-type fish exhibited multiple clusters of *TgBAC(neurod:*GFP)-positive cells along the intrapancreatic ducts (arrowheads in A′, C′). All images are projections of confocal z-stacks. Ventral views, anterior to the top right. Dashed lines delineate the pancreas. Scale bars, 100 µm. (G–L) Wild-type and mutant larvae were sorted at 5 dpf based on their intrahepatic ductal system phenotypes (as assessed by the pattern of *Tg(Tp1bglob*:GFP) expression) and raised separately. At 2 (G, J), 3 (H, K), and 4 weeks (I, L) of age, 2 wild-type and 2 mutant fish were fixed for immunostaining. Cryosections were stained with 2F11 (red) and anti-GFP (green) antibodies. In the liver, whereas the intrahepatic biliary network continued to expand between 2 and 4 weeks in wild-types (G–I), the biliary cells in *sox9b* mutants remained clustered and never assumed normal morphogenesis (J–L). Moreover, the expression of *Tg(Tp1bglob:*GFP) largely overlapped with 2F11 labeling in wild-type livers (G–I). In contrast, in *sox9b* mutant livers, we observed an increasing number of cells that were labeled by 2F11, but did not express *Tg(Tp1bglob:*GFP) (J–L). These cells formed large clusters by 4 weeks of age (L). All images are projections of confocal z-stacks. Sagittal sections, anterior to the top. Dashed lines in (G–L) delineate the liver. Li, liver; Pa, pancreas. Scale bar, 20 µm.
(TIF)

Figure S3 *sox9b* mutants show defects in bile secretion and transport as assessed by BODIPY-FL analog feeding. (A–B) Fluorescent micrographs of 7 dpf live wild-type (A) and *sox9b* mutant (B) larvae after BODIPY feeding showing lack of filling of the gallbladder (arrowhead) in *sox9b* mutants (B). Lateral views, anterior (A) to the left. (C–F) Confocal images of *Tg(Tp1bglob:H2B-mCherry)* wild-type (upper panel) and *sox9b* mutant (lower panel) larvae showing morphological and functional defects of both intrahepatic (D) and intrapancreatic (F) ductal networks compared to wild-type (C and E). In the mutants, both intrahepatic and intrapancreatic ducts appear to be dilated (D and F). Fluids (bile or pancreatic juice) also appear to accumulate in the pancreatic tail (arrows, F). Dashed squares represent areas shown in higher magnification for intrahepatic (C′–D′) and intrapancreatic (E′–F′) ducts in wild-types (upper panel) and *sox9b* mutants (lower panel). *sox9b* mutants showed defects in bile canaliculi (comparing arrowheads in C′, D′) and terminal pancreatic ducts (comparing arrowheads in E′, F′). 9 larvae were analyzed for each genotype. (C–F) All images are projections of confocal z-stacks. (C–F) Lateral views, anterior (A) to the left. Dashed lines in E and F outline the pancreas. g, gut; Li, liver. Scale bars, 20 µm.
(TIF)

Figure S4 The intrahepatic biliary cells in *sox9b* mutants are more susceptible to Notch signaling inhibition. (A–D) Confocal images of livers in *Tg(Tp1bglob:VenusPest); Tg(Tp1bglob:H2B-mCherry)* wild-type (A, C) and *sox9b* mutant (B, D) larvae treated with DMSO (A–B) or 50 µM DAPT (C–D) from 106 to 154 hpf. DAPT treatment caused an increase in the relative proportion of *Tg(Tp1bglob:*H2B-mCherry)-single positive cells in all the animals, yet *sox9b* mutants exhibited a more severe increase compared to wild-type larvae. In DAPT-treated wild-type larvae (C), loss of Notch activity was more prominent in the region proximal to the extrahepatic duct (p, left side of yellow line), whereas the distal biliary cells still maintained *Tg(Tp1bglob:*VenusPest) expression (d, right side of the yellow line). (A–D) All images are projections of confocal z-stacks. Ventral views, anterior (A) to the top. Dashed lines outline the liver. Scale bar, 50 µm. (E) Percentages (average±SEM) of *Tg(Tp1bglob:*H2B-mCherry)-single positive cells relative to the total number of *Tg(Tp1bglob:*H2B-mCherry)-expressing cells. 10 DMSO control and 14 DAPT-treated larvae were analyzed for each genotype. Asterisks indicate statistical significance compared to equally-treated wild-type larvae: ***, p<0.005; ******, p<0.000005.
(TIF)
