# Supplementary figures and images for: sox9b Is a Key Regulator of Pancreaticobiliary Ductal System Development
Source: PLoS Genet. 2012 Jun 14;8(6):e1002754. doi: 10.1371/journal.pgen.1002754 (PMC3375260; doi:10.1371/journal.pgen.1002754)

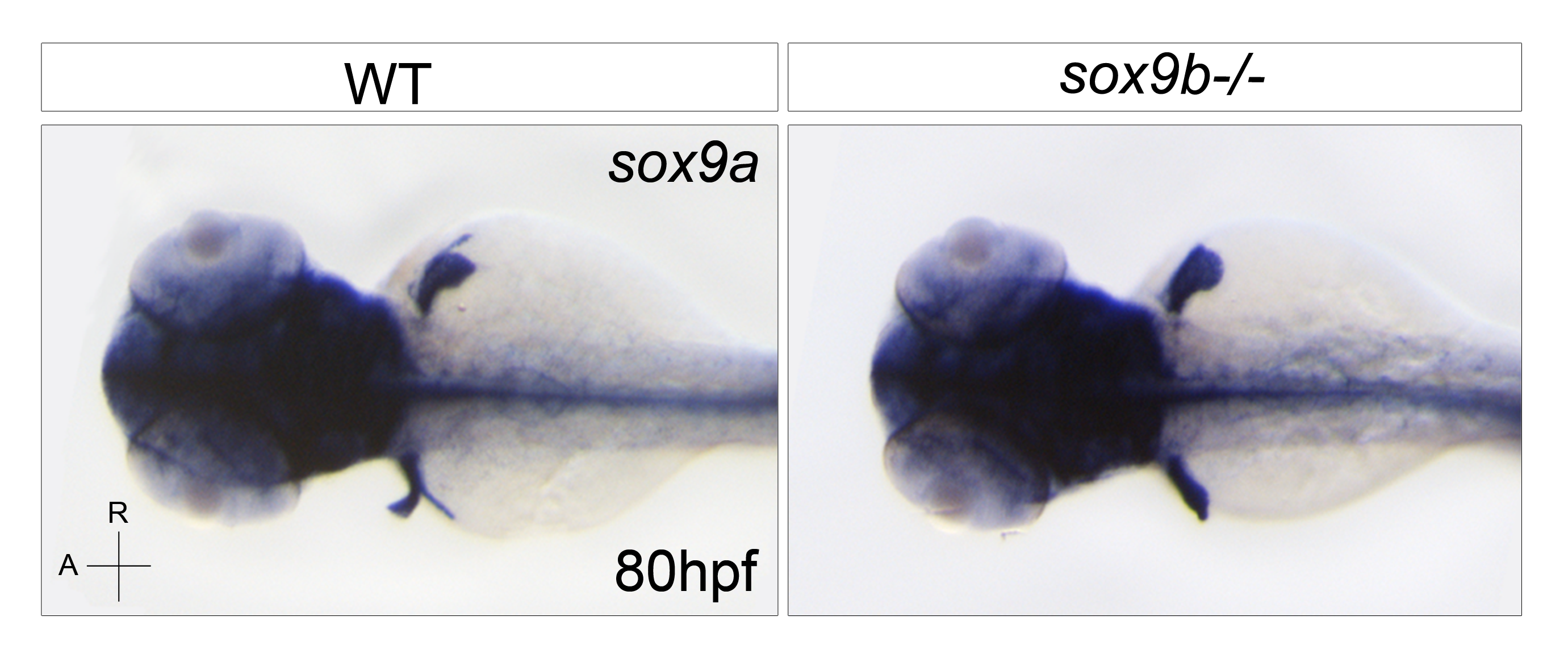

Supplement: Figure S1 — sox9a in situ hybridization in wild-type and sox9b mutant larvae at 80 hpf. sox9a does not appear to be expressed in endodermal tissues in wild-type or sox9b mutant animals. Dorsal views, anterior (A) to the left. (TIF) [file pgen.1002754.s001.tif]

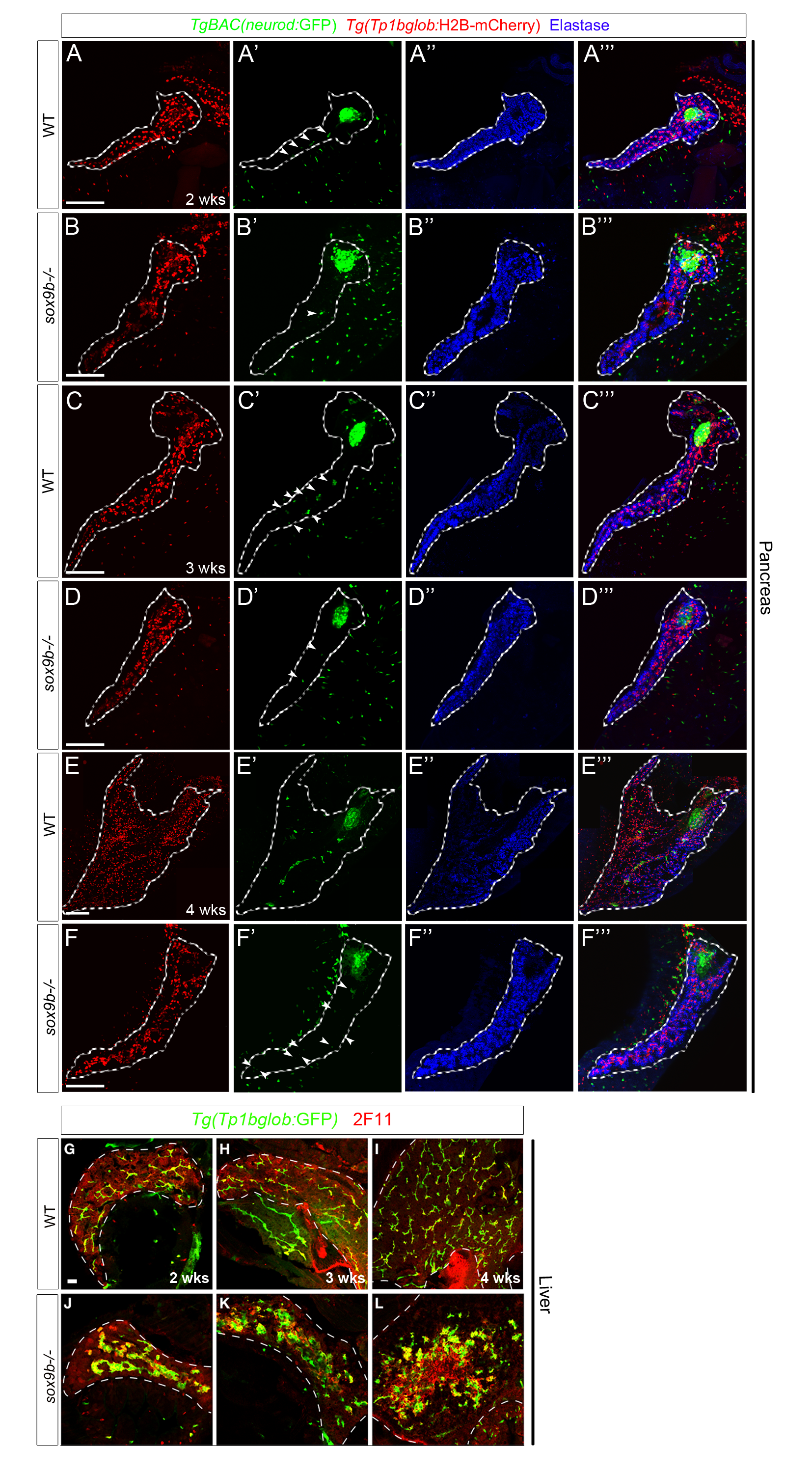

Supplement: Figure S2 — Time course analyses of the pancreas (A–F) and liver (G–L) in wild-type and sox9b mutant larvae at 2, 3, and 4 weeks. (A–F) Tg(Tp1bglob:H2B-mCherry);TgBAC(neurod:GFP) double transgenic fish from sox9b heterozygote incrosses were raised together and 40 of them were fixed and genotyped at each time point. The pancreas, along with the gut, of 6 to 11 wild-type or sox9b homozygous mutant fish were dissected, stained with anti-dsRed (red), anti-GFP (green) and anti-Elastase (blue) antibodies and mounted for confocal imaging. Whereas wild-type pancreata showed complex intrapancreatic duct networks at 2 and 3 weeks of age with several main pancreatic ducts (A, C), sox9b mutant pancreata failed to expand and ductal cells stayed in clusters (B, D). At 4 weeks of age, the morphological differences between wild-type and sox9b mutant pancreata were even more obvious: wild-type pancreata spread over the gut starting to form lobes whereas sox9b mutant pancreata were still primitive in appearance (E, F). The defect in pancreatic growth in sox9b mutant fish is associated with a global growth retardation of the fish as indicated by the smaller size of the fish and the frequent occurrence of an unlooped gut at 4 weeks (data not shown). In addition to pancreatic duct morphological defects, sox9b mutant fish showed a deficiency in secondary islet formation as assessed by TgBAC(neurod:GFP) expression (arrowheads in B′, D′, F′). However, at the equivalent stages, wild-type fish exhibited multiple clusters of TgBAC(neurod:GFP)-positive cells along the intrapancreatic ducts (arrowheads in A′, C′). All images are projections of confocal z-stacks. Ventral views, anterior to the top right. Dashed lines delineate the pancreas. Scale bars, 100 µm. (G–L) Wild-type and mutant larvae were sorted at 5 dpf based on their intrahepatic ductal system phenotypes (as assessed by the pattern of Tg(Tp1bglob:GFP) expression) and raised separately. At 2 (G, J), 3 (H, K), and 4 weeks (I, L) of age, 2 wild-typ [file pgen.1002754.s002.tif]

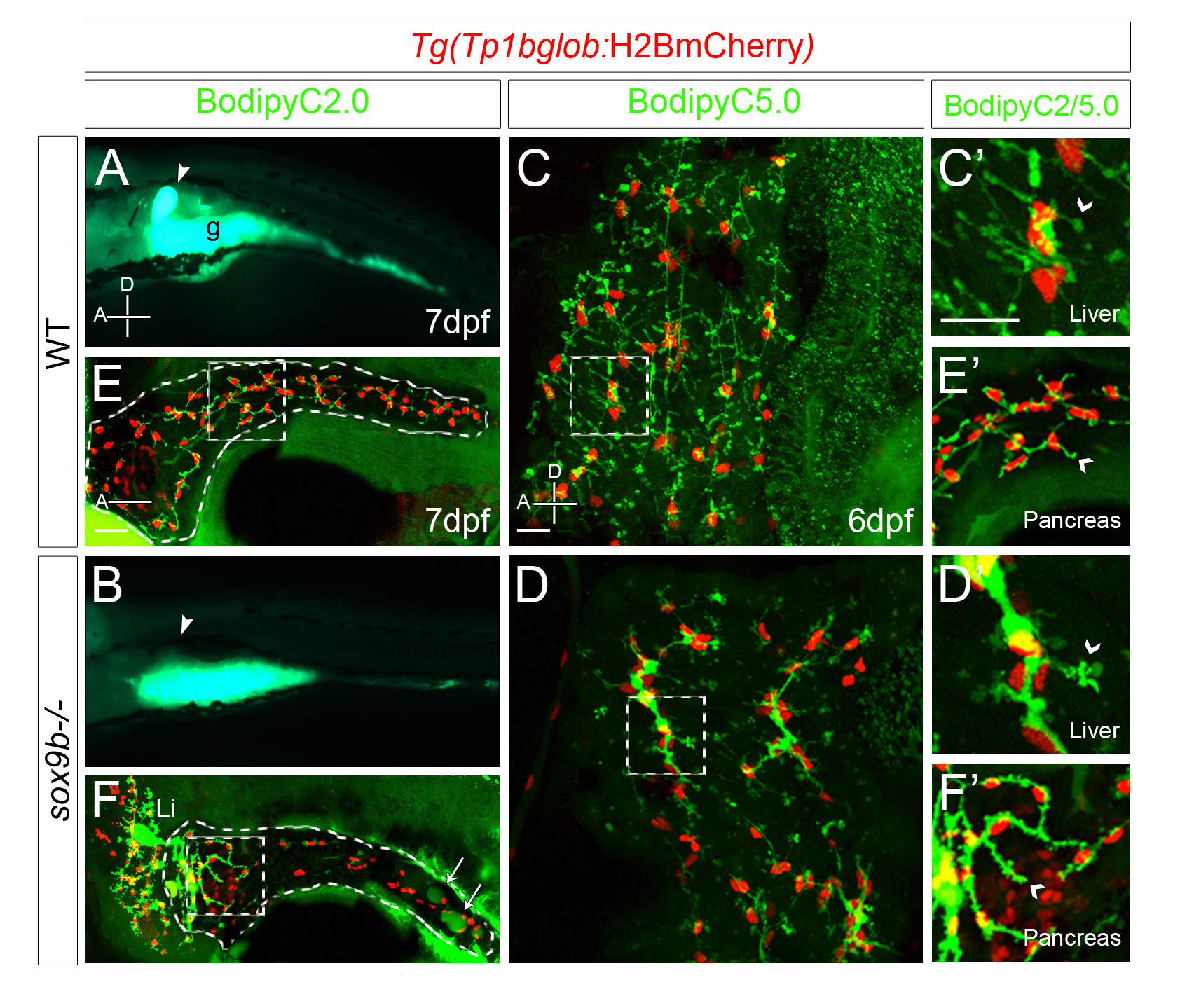

Supplement: Figure S3 — sox9b mutants show defects in bile secretion and transport as assessed by BODIPY-FL analog feeding. (A–B) Fluorescent micrographs of 7 dpf live wild-type (A) and sox9b mutant (B) larvae after BODIPY feeding showing lack of filling of the gallbladder (arrowhead) in sox9b mutants (B). Lateral views, anterior (A) to the left. (C–F) Confocal images of Tg(Tp1bglob:H2B-mCherry) wild-type (upper panel) and sox9b mutant (lower panel) larvae showing morphological and functional defects of both intrahepatic (D) and intrapancreatic (F) ductal networks compared to wild-type (C and E). In the mutants, both intrahepatic and intrapancreatic ducts appear to be dilated (D and F). Fluids (bile or pancreatic juice) also appear to accumulate in the pancreatic tail (arrows, F). Dashed squares represent areas shown in higher magnification for intrahepatic (C′–D′) and intrapancreatic (E′–F′) ducts in wild-types (upper panel) and sox9b mutants (lower panel). sox9b mutants showed defects in bile canaliculi (comparing arrowheads in C′, D′) and terminal pancreatic ducts (comparing arrowheads in E′, F′). 9 larvae were analyzed for each genotype. (C–F) All images are projections of confocal z-stacks. (C–F) Lateral views, anterior (A) to the left. Dashed lines in E and F outline the pancreas. g, gut; Li, liver. Scale bars, 20 µm. (TIF) [file pgen.1002754.s003.tif]

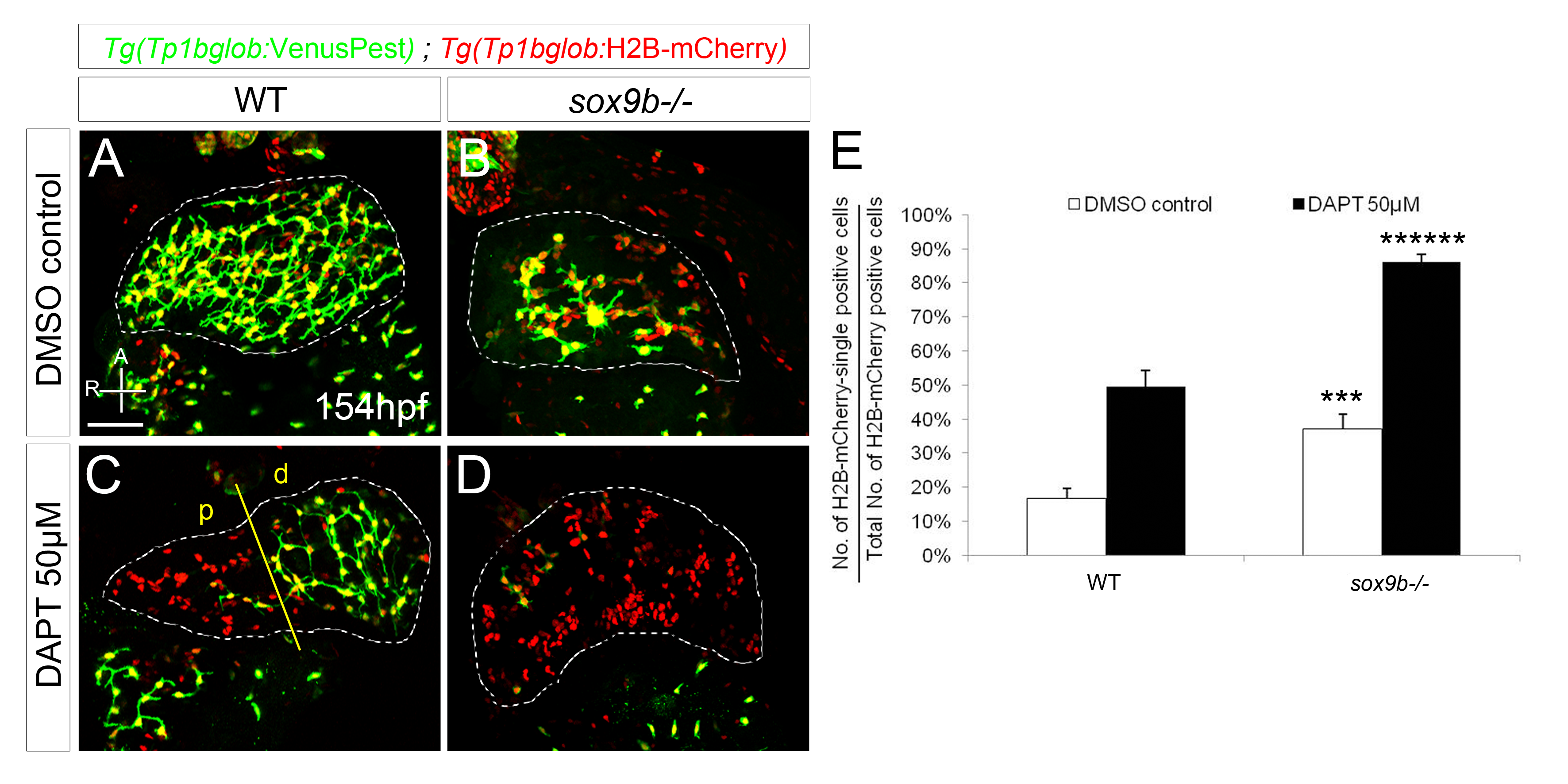

Supplement: Figure S4 — The intrahepatic biliary cells in sox9b mutants are more susceptible to Notch signaling inhibition. (A–D) Confocal images of livers in Tg(Tp1bglob:VenusPest); Tg(Tp1bglob:H2B-mCherry) wild-type (A, C) and sox9b mutant (B, D) larvae treated with DMSO (A–B) or 50 µM DAPT (C–D) from 106 to 154 hpf. DAPT treatment caused an increase in the relative proportion of Tg(Tp1bglob:H2B-mCherry)-single positive cells in all the animals, yet sox9b mutants exhibited a more severe increase compared to wild-type larvae. In DAPT-treated wild-type larvae (C), loss of Notch activity was more prominent in the region proximal to the extrahepatic duct (p, left side of yellow line), whereas the distal biliary cells still maintained Tg(Tp1bglob:VenusPest) expression (d, right side of the yellow line). (A–D) All images are projections of confocal z-stacks. Ventral views, anterior (A) to the top. Dashed lines outline the liver. Scale bar, 50 µm. (E) Percentages (average±SEM) of Tg(Tp1bglob:H2B-mCherry)-single positive cells relative to the total number of Tg(Tp1bglob:H2B-mCherry)-expressing cells. 10 DMSO control and 14 DAPT-treated larvae were analyzed for each genotype. Asterisks indicate statistical significance compared to equally-treated wild-type larvae: ***, p<0.005; ******, p<0.000005. (TIF) [file pgen.1002754.s004.tif]
